# Late winter under ice pelagic microbial communities in the high Arctic Ocean and the impact of short-term exposure to elevated CO_2_ levels

**DOI:** 10.3389/fmicb.2014.00490

**Published:** 2014-09-29

**Authors:** Adam Monier, Helen S. Findlay, Sophie Charvet, Connie Lovejoy

**Affiliations:** ^1^Département de Biologie, Québec Océan and Institut de Biologie Intégrative et des Systèmes, Université LavalQuébec, QC, Canada; ^2^Takuvik Joint International Laboratory (CNRS UMI-3376), Université LavalQuébec, QC, Canada; ^3^Plymouth Marine LaboratoryPlymouth, UK

**Keywords:** ocean acidification, Arctic Ocean, community structure, phylogenetic diversity, NRI, bottle effect

## Abstract

Polar Oceans are natural CO_2_ sinks because of the enhanced solubility of CO_2_ in cold water. The Arctic Ocean is at additional risk of accelerated ocean acidification (OA) because of freshwater inputs from sea ice and rivers, which influence the carbonate system. Winter conditions in the Arctic are of interest because of both cold temperatures and limited CO_2_ venting to the atmosphere when sea ice is present. Earlier OA experiments on Arctic microbial communities conducted in the absence of ice cover, hinted at shifts in taxa dominance and diversity under lowered pH. The Catlin Arctic Survey provided an opportunity to conduct *in situ*, under-ice, OA experiments during late Arctic winter. Seawater was collected from under the sea ice off Ellef Ringnes Island, and communities were exposed to three CO_2_ levels for 6 days. Phylogenetic diversity was greater in the attached fraction compared to the free-living fraction *in situ*, in the controls and in the treatments. The dominant taxa in all cases were Gammaproteobacteria but acidification had little effect compared to the effects of containment. Phylogenetic net relatedness indices suggested that acidification may have decreased the diversity within some bacterial orders, but overall there was no clear trend. Within the experimental communities, alkalinity best explained the variance among samples and replicates, suggesting subtle changes in the carbonate system need to be considered in such experiments. We conclude that under ice communities have the capacity to respond either by selection or phenotypic plasticity to heightened CO_2_ levels over the short term.

## Introduction

Since the industrial revolution, it has been estimated that the world's oceans have absorbed ~25% of all carbon dioxide (CO_2_) emitted into the atmosphere (Sabine et al., 2004; Sarmiento et al., 2010). This continual and rapid uptake of CO_2_ into the oceans is resulting in a shift in the ocean carbonate chemistry and a reduction in ocean pH, a process that has been termed ocean acidification (Caldeira and Wickett, 2003; Raven et al., 2005; Doney et al., 2009). The Polar Oceans naturally are sinks to CO_2_ because CO_2_ is more soluble in cold water. The Arctic Ocean is believed to be more at risk to accelerated ocean acidification because of the freshwater influence from sea ice and through river inputs (Steinacher et al., 2009). There is already evidence of low pH and undersaturated conditions in some areas of the Arctic Ocean (Bates et al., 2009; Mathis et al., 2012), and during winter, microbial respiration acts to further elevate CO_2_ in the surface waters under sea ice (Sabine et al., 2004; Sarmiento et al., 2010; Miller et al., 2011).

Microbial processes are responsible for biogeochemical cycling and functioning of marine ecosystems (Caldeira and Wickett, 2003; Raven et al., 2005; Azam and Malfatti, 2007; Doney et al., 2009) and the Arctic Ocean is no exception, with active bacterial production occurring even in the dark of winter (Garneau et al., 2008; Steinacher et al., 2009). Although ice covered regions appear to have lower bacterial production and abundance than ice-free areas, they also have much higher ratios of bacteria to phytoplankton production (Rich et al., 1997; Bates et al., 2009; Mathis et al., 2012), implying high levels of bacterial respiration. There are few reports of microbial community composition and activity during the Arctic winter, although a number of bacterial taxa remain active over the winter (Krembs et al., 2002; Junge et al., 2004; Galand et al., 2008, 2013). However, the majority of studies to date have investigated microbial processes during the spring, summer and early autumn when the Arctic Ocean is more accessible and under ice winter bacterial communities and processes remain understudied. The continued remineralization of carbon by pelagic bacteria throughout the winter under the sea ice is believed to contribute a significant fraction of the CO_2_ to the water column (Miller et al., 2011). On a seasonal cycle, the addition or removal of CO_2_ by microbes contributes significantly to the mediation of ocean acidification, which can have implications for the timing and rate of acidification. Therefore, understanding how a shift in climate or ocean acidification might impact bacterial abundance, community or activity is important for determining the feedbacks on biogeochemical cycling.

In the majority of recent ocean acidification experiments, bacterial abundance seems to be unaffected by CO_2_ levels (Rochelle-Newall et al., 2004; Grossart et al., 2006; Allgaier et al., 2008). The response of bacterial production to elevated CO_2_ conditions is not uniform; but most studies show an increase in bacterial production when exposed to elevated CO_2_ (Liu et al., 2010). The effect of acidification on bacterial community composition is variable; with either no change with increasing CO_2_ (Mühling et al., 2006; Allgaier et al., 2008; Tanaka et al., 2008), a shift to a community representing an “unhealthy” system, for example in coral-associated bacteria (Thurber et al., 2009), or an indirect effect with community changes associated with a phytoplankton bloom (Allgaier et al., 2008).

Studies addressing the effect of CO_2_ levels in Arctic Seas are particularly rare but a recent mesocosm experiment in an ice-free Arctic fjord reported that Gammaproteobacteria were the only taxon that showed significant changes with increasing CO_2_. In addition, the relative abundance of 15 rare taxa was significantly correlated with increasing CO_2_ concentration (Roy et al., 2013). The change in relative abundance of these rare taxa hints at potential shifts in taxa dominance or diversity, but overall the results were difficult to interpret, with major differences between the natural communities of the fjord and those in the mesocosm.

Our study aimed to investigate the direct effects of ocean acidification on under-ice Arctic pelagic bacteria, using culture-independent, high-resolution molecular techniques to not only assess changes in community structure, but also investigate changes in phylogenetic taxa dominance and diversity between both the particle-attached and the free-living communities. Since the natural community was also monitored before and after the experiment, our analysis also provided an opportunity to explore the influence of confinement, the so-called “bottle effect” on microbial communities.

## Materials and methods

### Field and experimental procedures

#### Ice base

From 15th March to 30th April 2010, the Catlin Ice Base (CIB) was situated at N 78° 43.11′, W 104° 47.44′ on a region of flat, first year sea ice, which extended east to west from Ellef Ringnes Island seaward (Supplementary Figure 1). The sea ice in the area was between 1.5 and 1.7 m thick. The water depth under the ice was not precisely known, although the maximum depth of the wire on the winch used for obtaining water samples was roughly 230 m, and this did not reach the bottom. The Canadian Hydrographic Service Chart #7953 (last updated 17/03/1972) suggests that the area is between soundings of 290 and 420 m.

#### Seawater collection, experimental set up and sampling

Seawater was collected, using a 10 L Niskin water bottle through a 1.3 × 1.1 m hole in the sea ice, from 25 m depth and placed initially into three sterilized 25 L water containers on the 19th April 2010. In each container the carbonate chemistry was manipulated using HCl and NaHCO_3_ based on measurements of pH and alkalinity (Table 1) and calculated using the Seacarb program (Lavigne and Gattuso, 2010) as detailed in the EPOCA Guide to Best Practices (Riebesell et al., 2010). The carbonate system was manipulated so that there were three atmospheric CO_2_ concentration scenarios: “ambient-CO_2_” (~400 ppm), “mid-CO_2_” (~880 ppm), and “high-CO_2_” (~1800 ppm). After the manipulations were made, seawater was decanted into three replicate 2 L incubation bottles (Thermo Scientific Nalgene polycarbonate narrow-mouth bottle) for each treatment. The incubation bottles were maintained at ambient seawater conditions by suspending them on a line through a hole in the sea ice (the hole was ~1 m^2^). The bottles were held 1.5 m under the ice for the duration of the experiment (6 days). The hole was maintained accessible (i.e., minimizing ice growth) by covering with wooden boards at night-time and removing ice during the day. Because of limited sample processing capacity, the three comparative *in situ* communities were from separate 10 m deep seawater samples; two samples were collected prior to OA experiments on 18/04/2010 and 22/04/2010 and one just after the experiment on 04/26/2010 (Table 1) for both free-living and attached fractions.

**Table 1:** **Environmental conditions from the water column under the sea ice over the same period as the ocean acidification experiment (before, during and after): salinity, temperature, dissolved inorganic carbon (DIC), and alkalinity were measured alongside chlorophyll, ammonium, phosphate, nitrate and silicate**.

	**18/04/10**	**22/04/10**	**26/04/10**
Salinity	30.31 (±0.22)	30.36 (±0.26)	30.33 (±0.12)
Temp (°C)	−1.652 (±0.002)	−1.650 (±0.003)	−1.654 (±0.006)
Alkalinity (μmol kg^−1^)	2140.5 (±4.8)	2143.3 (±6.8)	2141.1 (±2.2)
DIC (μmol kg^−1^)	2023.5 (±0.6)	2020.8 (±2.1)	2023.9 (±1.3)
pH_T_	8.137 (±0.012)	8.152 (±0.026)	8.137 (±0.007)
HCO^−^_3_ (μmol kg^−1^)	1914.8 (±1.5)	1909.7 (±5.7)	1915.1 (±2.4)
CO^2-^_3_ (μmol kg^−1^)	88.7 (±2.5)	91.9 (±5.0)	88.9 (±1.6)
CO_2_ (μmol kg^−1^)	20.0 (±1.5)	19.2 (±4.3)	19.9 (±1.8)
*p*CO_2_ (μatm)	290.4 (±8.2)	279.6 (±16.9)	290.1 (±5.5)
Ω_Cal_	2.18 (±0.06)	2.25 (±0.13)	2.18 (±0.04)
Ω_Arg_	1.35 (±0.04)	1.40 (±0.08)	1.35 (±0.03)
Chlorophyll (μg L^−1^)	0.58 (±0.11)	0.69 (±0.06)	0.73 (±0.14)
Ammonium (μM)	0.26 (±0.24)	0.40 (±0.15)	0.22 (±0.06)
Phosphate (μM)	0.77 (±0.11)	0.70 (±0.12)	0.78 (±0.03)
Nitrate (μM)	1.04 (±0.15)	1.15 (±0.21)	1.18 (±0.28)
Silicate (μM)	6.70 (±1.11)	5.93 (±1.12)	6.31 (±0.55)
Daily Surface PAR (W m^−2^)	18.66	22.86	28.81

*pH_T_, bicarbonate ion (HCO^−^_3_), carbonate ion (CO^2−^_3_) and aqueous CO_2_ concentrations, pCO_2_ and saturation states of calcite (Ω_Cal_) and aragonite (Ω_Arg_) were calculated from the measured parameters using CO_2_sys. Values are mean ± 1 standard deviation of the surface water measurements (0.5, 3, 5, and 10 m). Daily surface PAR values were estimated from the NCEP/NCAR surface level downward solar radiation flux (W m^−2^; 6 h measurements); values are daily integrations*.

Temperature, salinity and pH were measured at the beginning and the end of the experiment in water samples taken from each of the incubation bottles (Table 2). Temperature and salinity were measured using a handheld WTW LF197 multi-meter with a Tetra con 325 electrode, and pH was measured using a handheld a Metrohm 826 pH meter and pH electrode (6.0228.000), and using Amp- and Tris- buffers, to give pH on total scale, following Dickson et al. (2007). Alkalinity was measured by collecting water samples in 250 mL borosilicate glass bottles with ground glass stoppers according to standard procedures detailed in Dickson et al. (2007). Samples were stored unfrozen and returned to Plymouth Marine Laboratory (UK) for analysis using the open-cell potentiometric titration method using an automated titrator (repeatability: max. ± 0.1% at alkalinity ~2300 μmol kg^−1^). Calibration was made using Dickson Certified Reference Materials. For community DNA, 2 L of seawater was collected at the end of the experiment, and was first passed through a 50 μm mesh then sequentially filtered onto a 3 μm polycarbonate filter (Millipore) and a Sterivex 0.2 μm filter (Millipore). Filters were stored in lysis buffer (50 mM Tris, 40 mM EDTA, 0.75 M sucrose) and kept in liquid nitrogen until extractions at Université Laval (Canada).

**Table 2:** **Environmental conditions for the experimental treatments for the duration of the experiment: salinity, temperature, pH (total), and alkalinity were measured; dissolved inorganic carbon (DIC), bicarbonate ion (HCO^−^_3_), carbonate ion (CO^2−^_3_) and aqueous CO_2_ concentrations, *p*CO_2_ and saturation states of calcite (Ω_Cal_) and aragonite (Ω_Arg_) were calculated using CO_2_sys**.

	**Control**	**Mid CO_2_**	**High CO_2_**
Salinity	30.2 (±0.2)	30.1 (±0.1)	30.2 (±0.0)
Temp (°C)	−1.65 (±0.0)	−1.65 (±0.0)	−1.65 (±0.0)
pH_T_	7.999 (±0.006)	7.719 (±0.011)	7.388 (±0.007)
Alkalinity (μmol kg^−1^)	2146.5 (±50.0)	2149.5 (±12.0)	2068.7 (±59.8)
DIC (μmol kg^−1^)	2072.6 (±50.0)	2150.1 (±13.3)	2161.1 (±62.4)
HCO^−^_3_ (μmol kg^−1^)	1977.7 (±48.0)	2057.6 (±12.5)	2025.9 (±58.6)
CO^2-^_3_ (μmol kg^−1^)	66.6 (±1.5)	36.2 (±0.8)	16.7 (±0.6)
CO_2_ (μmol kg^−1^)	28.4 (±1.0)	56.3 (±1.6)	118.5 (±3.7)
*p*CO_2_ (μatm)	412.0 (±13.5)	817.6 (±22.9)	1722.9 (±54.2)
Ω_Cal_	1.64 (±0.03)	0.89 (±0.02)	0.41 (±0.02)
Ω_Arg_	1.02 (±0.02)	0.55 (±0.01)	0.26 (±0.01)

*Values are mean ± 1 standard deviation*.

#### DNA extraction, amplification and sequencing

Community DNA was extracted using a salt (NaCl) extraction protocol (Aljanabi and Martinez, 1997) as modified in Harding et al. (2011) and stored at −20°C until shipment to Argonne National Laboratory where the DNA samples were amplified with identifier tags along with 16S primers covering the V3−V4 region (Prof. J. A. Gilbert, Argonne National Laboratory, USA), then cleaned using the MoBio Ultra Clean htp 96 well clean-up kit. The clean amplicons were then quantified using Invitrogen PicoGreen reagent and a plate reader. Different volumes of the amplicons were pooled together into one tube with a target of 10–35 ng of amplicon DNA. The aim was to have similar quantities from each of the samples. Total DNA was quantified using a Qubit fluorometer (Life Technologies). Amplicons were sequenced using the Genome Analyser IIx Illumina platform, on which a 12 × 150 cycle run, with a 12 bp barcode read followed by a 150 bp sequence read was conducted.

### Data analyses

#### Sequence processing

Illumina reads were first pre-processed through the MG-RAST quality-control pipeline (Meyer et al., 2008). The reads were retrieved from the MG-RAST server and were analyzed using QIIME v1.6 (Caporaso et al., 2010b). Putative chimeric products were detected using UCHIME v4.2 (Edgar et al., 2011) and were removed from subsequent analyses as well as reads shorter than 75 nucleotides. Resulting reads were then clustered into operational taxonomic units (OTUs) with UCLUST, part of the USEARCH v6.0 software suite (Edgar, 2010), using a 97% sequence identity cutoff. OTUs consisting of only a single read in the entire data set (singletons) were discarded. In addition, one sample triplicate (Mid-CO_2_, attached fraction, replicate 2) was composed of less than 100 reads passing all filtering steps and was removed from the study. Our dataset was eventually composed of 7,182,447 high quality reads, clustered into 29,753 OTUs distributed across 23 distinct samples (see Supplementary Table 1 for workflow). Catlin OA sequence data are publicly available via MG-RAST (http://metagenomics.anl.gov) under the sample identifiers mgs29275–29294.

#### Taxonomic classifications

The OTU representative reads were taxonomically classified using the Ribosomal Database Project (RDP) multi-classifier tool (Wang et al., 2007), with a 50% assignment confidence cutoff. RDP release 9, with its associated hierarchical taxonomic classification (Cole et al., 2007), was used as the reference sequence database.

#### Phylogenetic diversity metrics

To compute phylogenetic beta-diversity measures, we reconstructed an approximate maximum-likelihood phylogenetic tree with FastTree v2.1 (Price et al., 2010) using GTR model and pseudocounts in “accurate mode” (-mlacc 2 -slownni); the phylogenetic tree reconstruction was based on a multiple sequence alignment of all OTU representatives generated with pyNAST (Caporaso et al., 2010a). Weighted (normalized) and unweighted UniFrac distances (Lozupone and Knight, 2005) among the different microbial communities, as well as phylogenetic diversity rarefaction curves were all computed based on the OTU approximate maximum-likelihood phylogenetic tree. Clustering of UniFrac distances was performed using the unweighted pair group method with arithmetic mean (UPGMA) algorithm, and cluster robustness was assessed using 1000 jackknife replicates (on 75% subsets).

We then investigated the variation in phylogenetic clustering between the different communities by computing net-relatedness index (NRI) (Webb et al., 2002). We reconstructed class-specific phylogenetic trees as above, for each of the sixteen dominant bacterial classes and the plastid 16S rRNA genes that were considered as a single class. The OTUs used for these trees were extracted from the multiple sequence alignments. Each phylogenetic tree was then analyzed with the R package “picante” v1.5 (Kembel et al., 2010) to compute abundance weighted mean pairwise distance (MPD) against a null model comprised of 999 randomized trees with taxa shuffling, leading to standardized metrics (*SES_MPD_*, corresponding to −1 × NRI).

#### Statistical analyses

Significances in taxonomic diversity differences and phylogenetic metrics among samples were assessed using analyses of variance (ANOVA) and Tukey's HSD *post-hoc* tests in the R environment (R Development Core Team; www.R-project.org).

For canonical correspondence analyses (CCA), we first conducted a correlation analysis on the environmental variables (Table 1) to avoid redundancy in the multivariate analyses. Correlations were detected between CO_2_ and *p*CO_2_ (of which we kept *p*CO_2_) and between CO_3_ and the calcite and aragonite saturation states (of which we kept CO_3_). Subsequently, two CCA were conducted to determine which environmental variables were correlated with changes among the microbial communities for the 12 most abundant phyla and for the Gammaproteobacteria. The OTU abundance data were transformed to relative proportions before conducting the CCA. Both correlation and ordination analyses were performed using PAST software (Hammer et al., 2009).

## Results

### Environmental parameters

To achieve these objectives, we sequenced the V3−V4 regions of the 16S small subunit rRNA gene (hereafter 16S) retrieved from natural microbial communities (“*in situ*”) and communities that were subjected to experimental manipulations. The experimental samples were put into 2 L containers and with three different CO_2_ concentrations including a bottle control “ambient-CO_2_”: (400 ppm), “mid-CO_2_” (880 ppm), and “high-CO_2_” (1800 ppm). Microbial assemblages were retrieved from two size fractions: small or “free-living” (0.2–3 μm), and large or “attached” (>3–50 μm).

The conditions within the experimental containers were maintained and stable over the 6 days. The salinity, temperature and total alkalinity in the control matched the *in situ* under-ice surface water conditions, however there were slight differences in other parameters, with lower pH and higher DIC and *p*CO_2_ conditions in the containers compared to *in situ* (Tables 1, 2).

### Phylogenetic diversity of natural and CO_2_-treated arctic microbial communities

**Figure 1:**
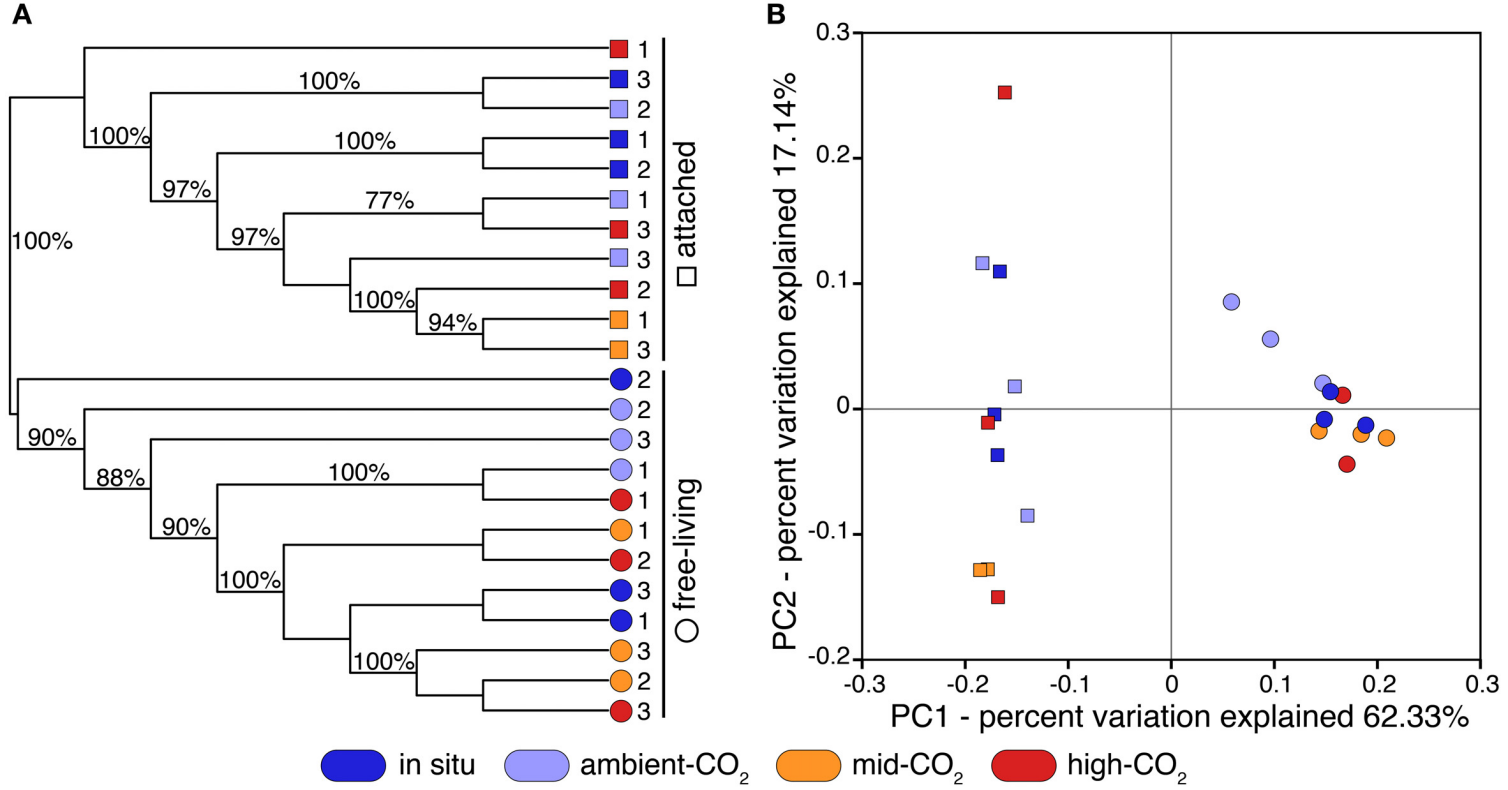
**(A)** Clustering of 23 Arctic pelagic microbial samples based on UPGMA of weighted and normalized UniFrac distances. Microbial communities were retrieved from two distinct size fractions (free-living: 0.2–3 μm; attached: >3 μm) and were sampled from natural conditions (*in situ*; dark blue) or from different CO_2_ concentration treatments: ambient-CO_2_ (400 ppm; clear blue), mid-CO_2_ (880 ppm; orange) and high-CO_2_ (1800 ppm; red). Samples are identified using replicate number. Cluster supports were computed using 1000 jackknife replicates. **(B)** Corresponding principal coordinate analysis (PCoA) using weighted and normalized UniFrac distances (PC: principal coordinate).

Our final 16S sequence dataset was composed of more than 7 million quality-controlled reads totaling ~30 k OTUs, across all samples (Supplementary Table 1). Clustering and principal coordinate analyses (PCoA) on both weighted and unweighted UniFrac distances indicated that size fraction (i.e., free-living vs. attached) mainly drove the phylogenetic beta-diversity across microbial assemblages (Figure 1 and Supplementary Figure 2, respectively). Weighted and unweighted distance clustering clearly separated the two size fractions, indicating their distinct phylogenetic compositions (Figure 1A and Supplementary Figure 2A). Moreover, the PCoA showed that phylogenetic compositions of communities from the attached fraction were remarkably more varied compared to those of the free-living communities (Figure 1B and Supplementary Figure 2B; along principal coordinate 2). Communities from the free-living fraction were more clustered along principal coordinates, indicating greater phylogenetic similarity among communities than those from the attached fraction.

There was little clustering by treatment with the replicates for the *in situ*, control, and the mid- and high-CO_2_ samples interspersed. The free-living fraction of the CO_2_-treated communities grouped into several supported clusters (Figure 1A), but with no pattern by treatment, and the clustering was not evident when using unweighted UniFrac distances (Supplementary Figure 2A). Two control samples were moderately distant from others when using weighted UniFrac distances (Figure 1B; along principal coordinate 1) but not unweighted ones (Supplementary Figure 2B). For the attached fraction, phylogenetic community composition of one of the high-CO_2_ replicates was very distant from all other samples, including from other high-CO_2_ samples, as determined by both weighted and unweighted PCoA analyses (Figure 1B and Supplementary Figure 2B). Also for the particle-attached size fraction, three samples from mid-CO_2_ and high-CO_2_ treatments significantly clustered together (Figure 1A and Supplementary Figure 2A).

**Figure 2:**
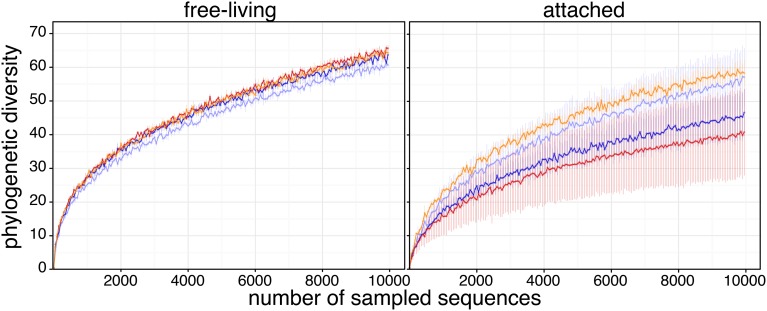
**Phylogenetic diversity rarefaction curves**. Phylogenetic diversity (mean ± s.e.m.) was measured as the sum of branch lengths of the 16S OTU phylogenetic tree for each rarefied sequence pool. Sequence datasets were rarefied to 10,000 sequences and were sampled using a step of 50 sequences; rarefaction sampling was reiterated 1000 times. Sample color code is as in Figure 1.

To test how acidification influenced the diversity of Arctic microbial assemblages, we computed the phylogenetic diversity (PD) of each sample. Using 1000 rarefaction iterations, no significant difference was found in PD among *in situ*, control and CO_2−_treated communities, for either size fractions (Figure 2). However, again there was much more variability within the attached fraction, compared to the free-living fraction.

### Acidification and detailed community taxonomic composition

**Figure 3:**
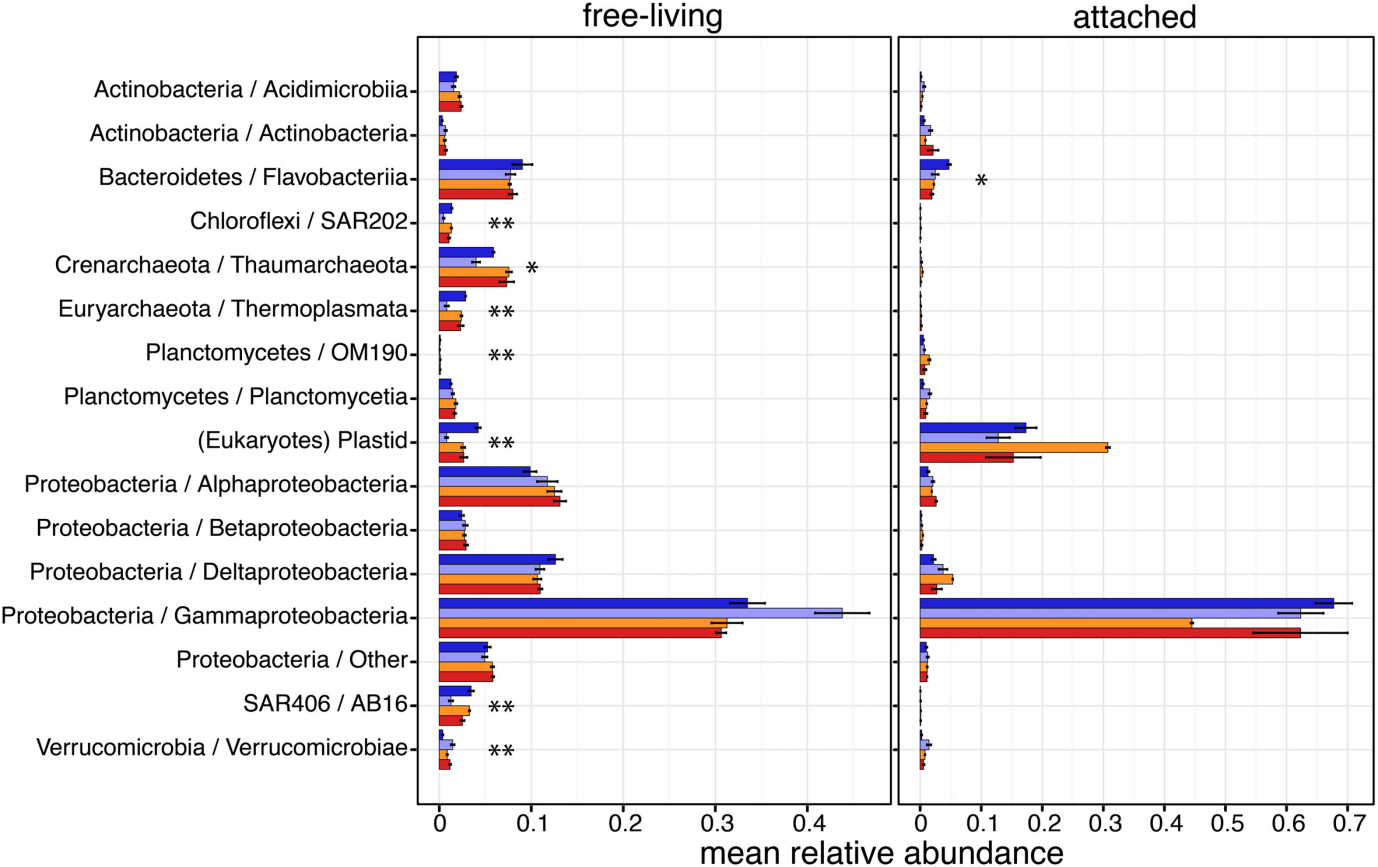
**Taxonomic compositions of Arctic pelagic microbial communities based on relative abundances (mean ± s.e.m.) of 16S sequences**. Only taxonomic groups with mean relative abundances >1% in at least one sample are shown. Sample color code is as in Figure 1. Asterisks indicate taxonomic groups with significantly different relative abundances between conditions (ANOVA; ^*^P < 0.1, ^**^P < 0.05).

The taxonomic composition of each microbial community was investigated by comparing relative abundances of 16S sequences among major Phyla and Classes of Proteobacteria, Actinobacteria and Planctomycetes, defined as those with a relative abundance >1% in at least one sample (Figure 3). OTUs classified as Gammaproteobacteria were by far the most abundant in both size fractions. Alphaproteobacteria, Betaproteobacteria and Deltaproteobacteria were more abundant in the free-living size fraction than in the attached. In the free-living fraction, seven of these major groups had significant differential relative abundance between *in situ* and CO_2_-treatments or across CO_2_-treatments (Figure 3 and Supplementary Table 2). Four of these were bacterial groups: SAR202 in the phylum Chloroflexi (*P* = 0.017), OM190 in the Planctomycetes (*P* = 0.024), SAR406 in the AB16 (*P* = 0.022) and Verrucomicrobiae in the Verrucomicrobia (*P* = 0.047). Both dominant archaeal groups were also found with differential relative abundances in the free-living fraction: Thaumarchaeota (*P* = 0.094) and Thermoplasmata in the Euryarchaeota (*P* = 0.026). Eukaryotic microbial phytoplankton detected by chloroplast 16S reads were also significantly affected by treatments (*P* = 0.011), being less abundant experimental containers compared to *in situ*. In the attached fraction, only OTUs classified as Flavobacteria in the Bacteroidetes (*P* = 0.079) were found with significant differential relative abundance, with fewer Flavobacteria reads in the containers compared to *in situ*.

**Figure 4:**
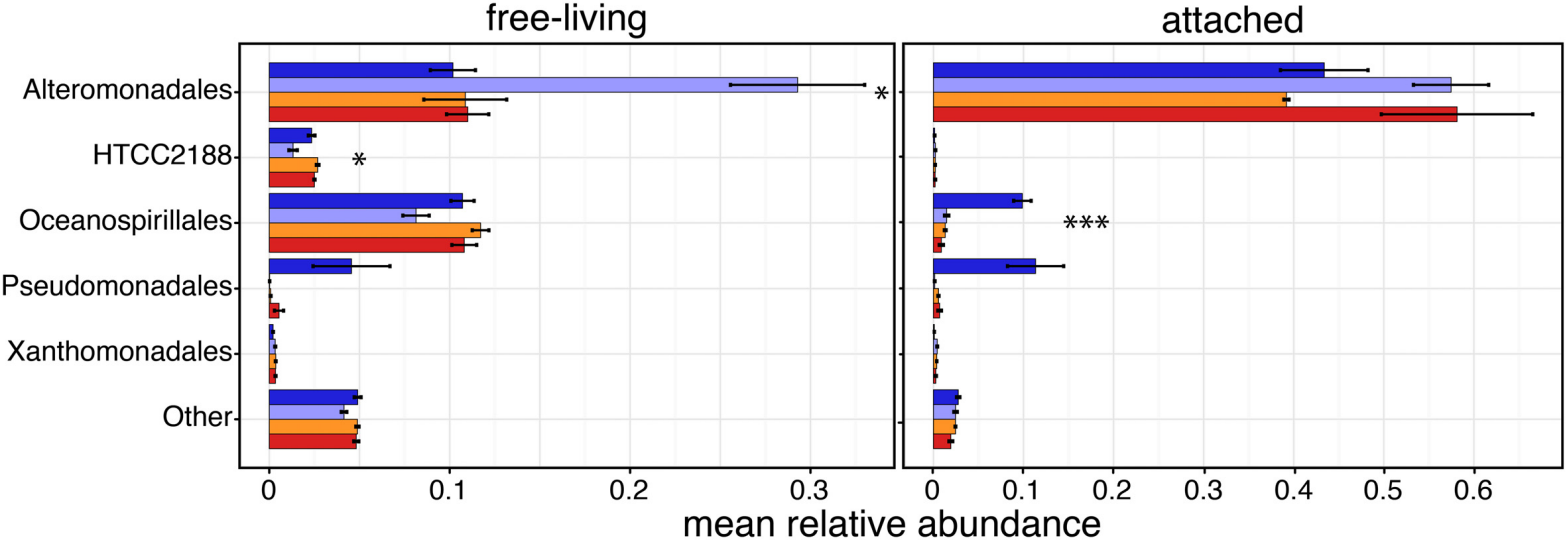
**Taxonomic compositions of the most abundant Gammaproteobacteria taxa (mean relative abundances >0.5% in at least one sample) based on relative abundances (mean ± s.e.m.) of 16S sequences**. Sample color code as in Figure 1. Asterisks indicate taxonomic groups with significantly different relative abundances between conditions (ANOVA; ^*^P < 0.1, ^***^P < 0.01).

Because Gammaproteobacteria were overall the most abundant class, we increased its taxonomic resolution to determine if the relative abundance of specific clades had been affected by the experimental conditions (Figure 4 and Supplementary Table 3). Alteromonadales constituted by far the most abundant lineage of Gammaproteobacteria in both size fractions, and were differentially abundant across samples in the free-living fraction (*P* = 0.054); free-living Alteromonadales were most abundant in the control samples, with a mean relative abundance of 0.29 (*SD* ± 0.12) compared to an *in situ* relative abundance of 0.1 (± 0.04). Interestingly, in both the CO_2_-treatments (mid- and high-CO_2_) values were similar to those *in situ* suggesting no or little “bottle effect”; free-living Alteromonadales, had similar relative abundances to those found in natural conditions (Supplementary Table 3). In the attached fraction, only Oceanospirillales were found to have significant differential relative abundances across conditions (*P* = 0.001); attached Oceanospirillales were quite abundant in the natural samples (0.098 ± 0.03), but their relative abundance was nearly 10-fold lower in the bottles, especially in the control (0.014 ± 0.008) and CO_2_-treated (e.g., mid-CO_2_: 0.013 ± 0.002) conditions.

### Acidification effects on community phylogenetic structure

We next examined the effects of CO_2_-treatments on the phylogenetic structure of each dominant taxonomic group. To this end, we used the net relatedness index (NRI) as a proxy for phylogenetic clustering (Webb et al., 2002). For a given community, positive NRI reflects phylogenetic clustering, indicating that the community is composed of taxa more evolutionarily related than randomly assembled communities. Such clustering can be an indicator of habitat filtering, where environmental conditions favor taxa with specific ecological traits, implying phylogenetic relatedness among taxa. In contrast, negative NRI reveals phylogenetic overdispersion, which indicates that taxa are less related than expected by chance. This overdispersion can potentially reflect exclusion by competition, where a taxon outcompeted closely related organisms exhibiting similar ecological traits.

**Figure 5:**
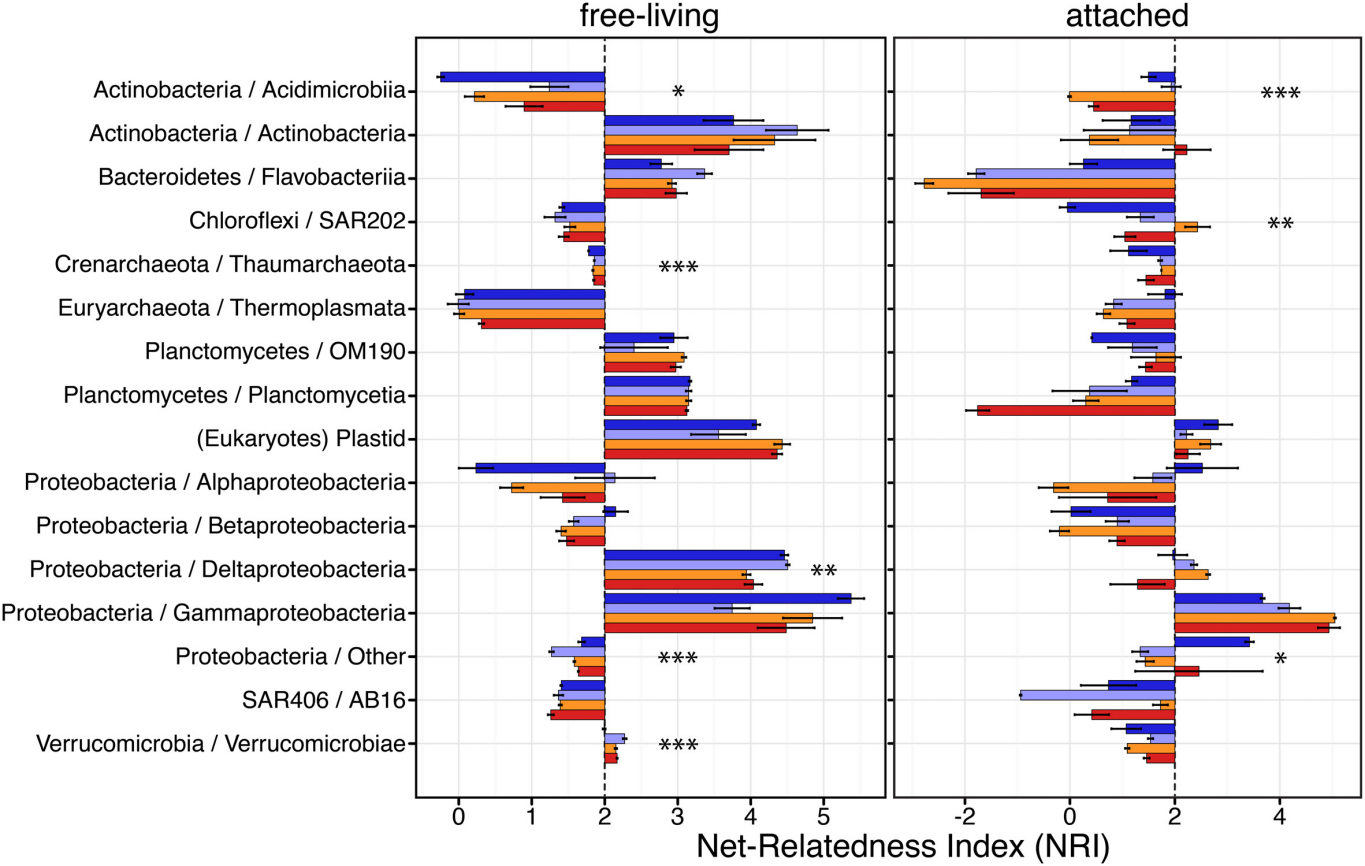
**Phylogenetic clustering of the Arctic pelagic microbial communities exposed to different CO_2_ concentrations**. Net-relatedness index (NRI, based on mean pairwise distances) measures branch-tip phylogenetic clustering. Sample color code as in Figure 1. Asterisks indicate taxonomic groups with significantly different NRI between conditions (ANOVA; ^*^P < 0.1, ^**^P < 0.05, ^***^P < 0.01).

Overall, we found that the different dominant taxonomic groups were phylogenetically structured, with a large majority of these exhibiting positive NRI values (Figure 5 and Supplementary Table 4). Five dominant taxonomic groups in the free-living fraction and three in the particle-attached had significant differences in their NRI across conditions. However, we found only a few cases of the phylogenetic structure of groups being impacted by CO_2_-treatment, specifically; Acidimicrobiia and Deltaproteobacteria. Acidimicrobiia was the only clade found to have significantly different NRI values in both size fractions. Free-living Acidimicrobiia communities were overdispersed in natural conditions but became clustered once isolated in bottles for control or CO_2_-treatements (Supplementary Table 4), with significant differences in NRI (*P* = 0.097). In contrast to their free-living counterparts, attached Acidimicrobiia became more overdispersed under higher CO_2_ levels (*P* = 0.005). We observed that the NRI of free-living Deltaproteobacteria communities decreased when exposed to higher CO_2_ levels. Bottled Verrucomicrobia communities from the small size fraction were found to have higher NRI values than *in situ* counterparts.

### Multivariate analysis

The shifts in community structure were investigated further using canonical correspondence analysis (CCA) of the OTUs alongside the carbonate system parameters measured in each treatment. The ordination distributions confirmed the difference in communities between the free-living (Supplementary Figure 3A) and attached (Supplementary Figure 3B) fractions. In terms of whole communities, the particle-attached bacteria showed higher variability among treatments, especially along axis 1 representing 87.54% of the variation, which was influenced by alkalinity, HCO_3_ and DIC. Conversely, the free-living communities of the treatments clustered together in ordination space along axis 1 (67.6% of the variation), which was largely affected by *p*CO_2_, CO^2−^_3_ and pH.

The attached Gammaproteobacteria communities were divided mostly according to alkalinity (axis 2; 22% of the variation) (Supplementary Figure 4A). Although axis 1 explained 77.9% of the variation, it primarily affected the separation of one outlier replicate of the high-CO_2_ treatment. The free-living Gammaproteobacteria communities were affected by changes in *p*CO_2_, DIC and HCO^−^_3_ (axis 1; 60.4% of the variation), mostly separating the mid-CO_2_ and high-CO_2_ treatments from the controls (Supplementary Figure 4B). The alkalinity also influenced these free-living Gammaproteobacteria communities, compared to the whole community (Axis 2; 34.6% of the variation).

## Discussion

### Community differences

The goal of our study was to assess the major components of the community changed, in terms of phylogenetic and taxonomic structure, in response to short-term pH modifications. Miller et al. (2011) reported rapid increases in *p*CO_2_ below Arctic ice and for this reason the experiments were carried out over 6 days to assess community response to such short-term increases in CO_2_. A major goal was to assess whether such increases would change the community structure, for example by selecting taxa more able to cope with more acidic conditions or if the community remained the same, implying more physiological plasticity among the microbes present. It would follow that if communities are plastic then these communities would remain taxonomically stable over the long term, despite anthropogenic increases of *p*CO_2_, provided these changes were of the same order. The NRI values provided us with a means of comparing the phylogenetic structure of the natural and CO_2_-exposed communities. The advantage of this approach is that it is independent from any “taxonomic lexicon,” that is, the phylogenetic information is used directly rather than mapping OTUs onto taxonomic information. Using NRI, we could determine and test for the fluctuations in phylogenetic structure across natural, control and CO_2_-exposed communities, rather than just relying on taxonomic nomenclature.

The major differences detected were between the attached and free-living communities, with significant differences in beta-diversity, taxonomy, and phylogenetic structure in free-living compared to the particle-attached communities (Figures 1, 2). Although in the open ocean the differences between attached and free-living communities vary, generally there are differences in coastal regions (Campbell and Kirchman, 2012; Mohit et al., 2014). Most studies in the Arctic are in more coastal waters, and it is clear that the particles represent a distinct environment in these regions (Garneau et al., 2006, 2009), as well as in the more offshore Beaufort Sea open ocean region (Kellogg and Deming, 2009; Ortega-Retuerta et al., 2013). Differences in taxonomic compositions of the particle-attached and free-living communities are likely dependent on the origin and age of particles, which influences their physical properties and organic content (Pinhassi et al., 1999; Ortega-Retuerta et al., 2013). In terms of alpha-diversity, the Arctic free-living communities were more diverse than the particle-attached assemblages (Figure 2). Interestingly Kellogg and Deming (2009) reported lower diversity for attached than free-living communities in the Laptev Sea, however in Kongsfjord, Svalbard and along the Beaufort Shelf particle attached bacteria were more diverse (Ortega-Retuerta et al., 2013; Sperling et al., 2013). This limited data would be consistent with regions away from direct river or coastal influence having less diverse particle types and as a consequence the attached bacteria are less diverse. Our data could therefore be indicative of the winter-time conditions when there is minimal river and/or coastal influence.

The attached communities in our study were also more over-dispersed than the free-living, which would be consistent with patchiness of particles within the environment and an artifact of the low volumes filtered. Each particle can be considered as a distinct habitat, with an initial founder community that is gradually replaced by a succession of bacteria that eventually results in the complete the degradation of the particle (Huston and Deming, 2002; Arnosti, 2008). The effect would force the net relatedness index of their combined communities to be over-dispersed. In contrast, the free-living organisms are affected by the environmental conditions at smaller scales and an incubation volume of 2 L would include more of the range of possible conditions selecting for a more closely related consortium. In sum, the attached communities may have been under-sampled, causing this lower overall diversity and strong variability. Neither community reached asymptotic and further high-throughput sampling may be required.

In general, for the Arctic marine microbial communities found in our study, the experimental CO_2_ enrichment had little influence on alpha- or beta-diversity. Other studies of ocean acidification on Arctic marine bacterioplankton communities similarly report that acidification does not appear to be a major factor influencing microbial community assemblages. A mesocosm acidification experiment on Arctic plankton communities carried out in Kongsfjord Svalbard revealed that the compositional change was explained by a concomitant phytoplankton bloom and temperature changes with little or no effect attributed directly to acidification levels (Roy et al., 2013). Sperling et al. (2013) suggested that any effect of acidification was indirectly a response to viral lysis of phytoplankton in the post-bloom phase under higher CO_2_ leading to the release of particulate organic substrates, which stimulated the bacterial community. Since we did not create a bloom in our experiment, nor monitor viral production, the two experiments are difficult to reconcile, except to state that acidification *per se* probably has little influence on bacterial communities in the Arctic over the short term. Indeed, although there was wide variability, the results of the CCA analysis suggest that in several cases alkalinity was a stronger driver than pH or *p*CO_2_ conditions (Supplementary Figures 3, 4). Alkalinity shifts could be important for the Arctic, as alkalinity is influenced by freshening and river inputs and flow, and thus requires further investigation.

For the free-living fraction there were more instances of changes between the *in situ* and control communities compared to the *in situ* and high CO_2_ treatments in our study, suggesting that a parameter in the higher CO_2_ treatments compensated for containerization. In contrast, there was no such pattern in the attached communities. Similarly, Allgaier et al. (2008) reported changes in the community of free-living bacteria but not in the larger size fraction in response to changes in acidification. Roy et al. (2013) also found a greater effect on the Gammaproteobacteria in the free-living fraction than with the particle-attached communities at higher *p*CO_2_ levels. A more pronounced response of the smaller free-living cells to the ambient conditions may be a consequence the greater influence of molecular diffusive processes on smaller cells (Yoshiyama and Klausmeier, 2008), whereas bacteria attached to particles are more likely to be surrounded by larger organic molecules (Malfatti and Azam, 2009) offering some protection from rapid chemical changes in the environment. However, the net effect on bacterial production is a separate question, which we did not address as we did not measure production. Interestingly, Grossart et al. (2006), found that in response to high *p*CO_2_, particle-attached communities were more active, in terms of protein production, than the free-living communities.

In most previous acidification experiments on microbial communities, changes in the bacterial communities were closely correlated with system productivity (Grossart et al., 2006; Allgaier et al., 2008; Roy et al., 2013). Additionally, Newbold et al. (2012) showed that acidification has a greater effect on picoeukaryotes than bacteria, supporting the idea that the changing *p*CO_2_ conditions have an indirect effect on bacterial communities through the re-structuring of phytoplankton communities. Additionally, acidification could hamper compositional shifts orchestrated by other environmental variables, as indicated by reports that the shift in Arctic bacterial community composition induced by glucose amendments is mitigated under acidified conditions (Ray et al., 2012). Therefore, although increased *p*CO_2_ had little direct impacts on the prokaryote taxonomic composition and phylogenetic structure, it could have indirect effects on microbial communities. The Catlin Arctic Survey also investigated the effects of OA at higher trophic levels and report that Pteropods were only weakly affected by acidification (Comeau et al., 2012) and copepod species showed contrasting responses to acidification, with the small *Oithona* species reacting more strongly than the larger *Calanus* (Lewis et al., 2013). The latter conducts deep vertical migrations through the water column under the winter ice whereas *Oithona* stay in the surface water layer. Hence *Calanus* and similarly the pteropods, are regularly exposed to varying concentrations of CO_2_ as they undergo their daily vertical migration, potentially making them less sensitive to changes in CO_2_ compared to the smaller non-vertically migrating *Oithona* (Lewis et al., 2013). These contrasting results across the food webs warrant further, collaborative investigations into whole ecosystem responses to OA. In addition, to predict the net effects of OA there is a need to evaluate the indirect responses of microbial communities perhaps by investigating many communities under a range of conditions including naturally high CO_2_ conditions related to increased photosynthesis or venting.

The minimal responses of bacterial communities to acidification reported here and elsewhere are likely the result of a tolerance of the microbial communities to changes in pH and CO_2_ established by the natural variability in ocean conditions (McNeil, 2010; McNeil et al., 2010; Gilbert, 2011). Daily pH variability is caused by tides, upwelling of deep waters and other ocean processes affecting mostly coastal systems and causing fluctuations of *p*H > 0.25 (Hofmann et al., 2011). While daily variability is less accentuated in the Antarctic (McNeil, 2010; Matson et al., 2011), there is limited comparable data for the Arctic. However formation and melting of ice can heighten the extent of pH and *p*CO_2_ excursions that microbial communities must contend with on a seasonal scale (Shadwick et al., 2013). The presence of sea ice induces a disequilibrium of CO_2_ exchanges between the water and the atmosphere, and during its formation expulsion of brines containing higher concentrations of Total Inorganic Carbon (TIC) into the underlying waters will cause an increase in their *p*CO_2_ content (Miller et al., 2011).

Moreover, a large part of the seasonal variability in the Arctic Ocean pH and *p*CO_2_ is a consequence of microbial activities; heterotrophic bacterial communities tend to increase *p*CO_2_ through respiration within and under the ice (Bates and Mathis, 2009; Nguyen et al., 2012). These biologically induced changes in pH and *p*CO_2_ take place on time scales much shorter than the projected OA due to climate change. The marine microorganisms behind these biogeochemical processes might already be adapted to respond to varying pH and *p*CO_2_ conditions. Indeed, pH and *p*CO_2_ also varies with depth, and hence microbial assemblages can contain similar bacterial lineages along a range of conditions (DeLong et al., 2006). This spatial and temporal variability is consistent with our results in the sense that the experimental CO_2_ treatments were likely not far outside the range of natural short-term variability experienced by the microbial community as a whole, thus precluding any marked taxonomic changes in the communities.

### Higher taxonomic resolution: gammaproteobacteria

Gammaproteobacteria were by far the most abundant taxa in both size fractions, consistent with the report of clone libraries of >3 and <3 μm fractions dominated by Gammaproteobacteria in the Laptev Sea (Kellogg and Deming, 2009). In contrast, Collins et al. (2010) found Alphaproteobacteria to dominate the microbial communities, mostly from the SAR11 clade during the same season as our study. Alonso-Sáez et al. (2012) found, using fluorescence *in situ* hybridizations combined with autoradiography, that under the ice Gammaproteobacteria appeared at lower concentrations but had a greater proportion of active cells compared to the numerically dominant SAR11 clade.

In contrast to reports from post-bloom conditions, where Gammaproteobacteria are the only free-living taxon affected by acidification (Roy et al., 2013), the Gammaproteobacteria relative abundance did not vary with containment or CO_2_-treatment in our experiment. Nonetheless, exploring higher taxonomic resolutions revealed some variability within the Gammaproteobacteria mostly in response to containment. Alternomonadales in the free-living community increased when contained in the incubation bottles, but was nearer the *in situ* proportion in the higher CO_2_ treatments. Alternomonadales are among the most easily isolated marine bacteria from enrichment cultures (Lovejoy personal observation) and the change in pH may have influenced quorum sensing in the containers. In the particle-attached fraction the Oceanospirillales were significantly less abundant in all experimental bottles compared to the *in situ* samples, and may have reflected a lack of suitable high molecular weight substrate for this group or the effect of the 50 micron pre-screening if the Oceanospirillales were associated with animals (La Riviere et al., 2013).

In terms of phylogenetic structure, Gammaproteobacteria did not show significant responses to either acidification or bottle containment. This resilience of Gammaproteobacteria to increased CO_2_ levels could be linked to maintenance of intracellular pH homeostasis at higher environmental CO_2_, as reported for several Gammaproteobacteria species (Zhao and Houry, 2010).

The *in situ* prevalence of free-living archaeal Thaumarcheota, in surface waters of the Arctic has been reported previously (Garneau et al., 2006; Comeau et al., 2011), although they represented <10% of the total prokaryote reads, compared to 18% in ice-covered surface waters of the Beaufort Sea (Alonso-Sáez et al., 2012). The lower relative abundance of Thaumarcheota in our study may have been related to substrate availability (Pedneault et al., 2014). Alternatively, methodological differences and primer bias cannot be discounted. The somewhat significant differences in the Thaumarcheota among experimental treatments appear to be more closely linked to containment than to the effects of CO_2_ (Supplementary Table 2).

### Bottle and multiple domain effects

Overall although we found a number of clusters with high support suggesting similarity of communities, there was no clear association with any particular treatment and we were not able to identify any factors driving community composition. In fact, this suggests that there were a finite number of community outcomes from the starting community and stochastic processes were predominant. Containment facilitates experimental manipulation of growth conditions and is a valuable tool for understanding how living organisms and communities react to specific variables, it can also be used to select for species with traits that enable growth under specific conditions (Zobell, 1943; Ferguson et al., 1984; Massana et al., 2001). However, in nature plankton are subjected to advective flow and diffusion in addition to biological processes and interactions. Since containment ceases the advective flow of cells, nutrients, and metabolites, it can cause bias by what is referred to as the “bottle effect” (Whipple, 1901). Here we compared *in situ* samples with ambiant-CO_2_, mid-CO_2_ and high CO_2_conditions, with the aim to control for the effect of experimental confinement on natural bacterial community composition. In fact, the majority of the significant changes in taxonomic abundance and phylogenetic structure, particularly for the attached communities, were in response to containment.

Interestingly more significant changes were observed between the *in situ* and the control (ambiant-CO_2_) bottles, with fewer differences detected between the *in situ* and mid- and high-CO_2_ treatments. Hence, we cannot rule out that higher CO_2_ may have influenced some unmeasured factors including viral infection or predation, which could increase within bottles, therefore mimicking the effects of a more open “natural” system, where growth and loss rates are balanced.

In contrast to other studies on Arctic waters, we have monitored the effects of acidification on Bacteria and Archaea communities independent of a phytoplankton bloom and suggest that some of the reported effects on bacterial communities were indirect and due to higher primary production and perhaps additional containment effects. While it has been suggested that OA should not have a significant impact on ocean biogeochemical processes, aside from calcification (Joint et al., 2010), the strong bottle effects suggests we are hampered in our ability to genuinely test the effect of acidification on microbial communities. However, despite this we were able to detect small shifts in phylogenetic structure and diversity that could have subtle effects on the maintenance of functionality in a complex community. New *in situ* tools need to be developed that could monitor such changes.

## Author contributions

All authors wrote the article; Helen S. Findlay designed and conducted field and experimental work; Adam Monier and Connie Lovejoy designed bioinformatics analyses; Adam Monier conducted bioinformatics analyses; Sophie Charvet conducted CCA analysis and provided logistic support:.

### Conflict of interest statement

The authors declare that the research was conducted in the absence of any commercial or financial relationships that could be construed as a potential conflict of interest.
