# Supplementary material for: Late winter under ice pelagic microbial communities in the high Arctic Ocean and the impact of short-term exposure to elevated CO2 levels
Source: Front Microbiol. 2014 Sep 29;5:490. doi: 10.3389/fmicb.2014.00490 (PMC4179612; doi:10.3389/fmicb.2014.00490)
Supplement: Supplementary file 1 [file DataSheet1.PDF]

Supplementary Material

Late winter under ice pelagic bacterial communities in the high Arctic Ocean and the impact of short-term exposure to elevated CO_2 levels

Adam Monier[1,2,§,†], Helen S. Findlay[3,†], Sophie Charvet[1,∞], Connie Lovejoy[1,2,*]

[1]Département de biologie, Québec Océan and Institut de Biologie Intégrative et des Systèmes (IBIS), Université Laval, Québec, QC, Canada; [2]Takuvik joint international laboratory (CNRS UMI-3376), Université Laval, Québec, QC, Canada;
[3]Plymouth Marine Laboratory, Plymouth, UK

[§]Present address: Biosciences, College of Life and Environmental Sciences, University of Exeter, Exeter EX4 4QD, UK
[∞]Present address: IOW Leibniz-Institut for Baltic Research Warnemünde, Germany
[†]Equal contributions
[*]**Correspondence:** Prof. Connie Lovejoy, Département de Biologie, Université Laval, Pavillon Alexandre Vachon, 1045 avenue de la medicine, Québec, QC G1V 0A6, Canada.
connie.lovejoy@bio.ulaval.ca

1. Supplementary Figures and Tables

Supplementary Table 1. Summary of sequence pre-processing and OTU clustering.

Supplementary Table 2. Relative abundance summary and statistics of the main taxonomic groups. Main groups were defined as those with mean relative abundances >1% in at least one sample.

Supplementary Table 3. Relative abundance summary and statistics of the main Gammaproteobacteria clades. Main Gammaproteobacteria clades were defined as those with mean relative abundances >0.5% in at least one sample.

Supplementary Table 4. NRI (net-relatedness index) summary and statistics for the most abundant clades (defined as those with mean relative abundances >1% in at least one sample).

Supplementary Figure 1. (A) Map of the Arctic Ocean, with area of sampling region highlighted by the black box. **(B)** Sampling location off Ellef Ringes Island marked by red circle.

Supplementary Figure 2. (A). UPGMA of unweighted UniFrac distance metrics. **(B).** PCoA of unweighted UniFrac distance metrics.

Supplementary Figure 3. Canonical correspondence analyses of the free-living fraction of the community for **(A)** the 12 most abundance phyla and **(B)** Gammaproteobacteria.

Supplementary Figure 4. Canonical correspondence analyses of the attached fraction of the community for **(A)** the 12 most abundance phyla and **(B)** Gammaproteobacteria.

1.1. Supplementary Tables

Supplementary Table 1. Summary of sequence pre-processing and OTU clustering.

Treatment	Replicate	Size fraction	MG-RAST identifier	Total raw reads	MG-RAST QC reads	Final QC reads	Final OTU
in situ	1	small	mgs29238	1,345,927	1,334,780	1,279,908	20,518
		large	mgs29239	15,553	15,465	14,958	2,245
	2	small	mgs29247	50,638	50,246	48,355	5,737
		large	mgs29248	836,809	830,998	803,555	10,973
	3	small	mgs29230	193,224	191,795	184,693	10,476
		large	mgs29231	178,887	177,521	166,047	4,279
ambient	1	small	mgs29275	167,840	166,900	160,863	9,915
		large	mgs29277	354,232	352,327	334,261	9,159
	2	small	mgs29278	1,592,544	1,584,413	1,522,257	18,691
		large	mgs29279	132,028	131,319	124,854	4,333
	3	small	mgs29280	142,294	141,352	135,705	8,939
		large	mgs29281	612,358	609,024	583,436	12,558
mid	1	small	mgs29289	220,629	219,397	211,356	11,097
		large	mgs29290	149,665	148,668	135,849	5,833
	2	small	mgs29291	186,485	185,299	177,354	10,825
		large	mgs29292	164	164	0	0
	3	small	mgs29293	367,860	365,636	349,875	14,105
		large	mgs29294	121,793	121,103	116,220	6,032
high	1	small	mgs29282	193,723	192,681	184,987	10,993
		large	mgs29283	29,809	29,674	28,740	727
	2	small	mgs29284	171,529	170,451	163,402	10,428
		large	mgs29285	117,488	116,753	107,639	5,222
	3	small	mgs29286	126,943	126,075	120,839	9,017
		large	mgs29288	239,365	238,179	227,294	6,517

Supplementary Table 2. Relative abundance summary and statistics of the main taxonomic groups. Main groups were defined as those with mean relative abundances >1% in at least one sample.

Taxonomic group	Size fraction	Sample	Mean relative abundance (s.d.)	ANOVA F-value	ANOVA P-value	Adjusted P-value (Tukey's HSD test)
Actinobacteria / Acidimicrobiia	free-living	in situ	0.018 (0.005)	1.639	0.255	-
		ambient	0.015 (0.005)			
		mid	0.022(0.004)			
		high	0.023 (0.004)			
	attached	in situ	0.0017 (0.0006)	1.599	0.273	-
		ambient	0.006 (0.005)			
		mid	0.003 (0.0002)			
		high	0.001 (0.001)			
Actinobacteria / Actinobacteria	free-living	in situ	0.003 (0.0009)	0.857	0.501	-
		ambient	0.006 (0.004)			
		mid	0.006 (0.003)			
		high	0.007 (0.003)			
	attached	in situ	0.006 (0.003)	0.49	0.699	-
		ambient	0.017 (0.009)			
		mid	0.008 (0.0008)			
		high	0.0211 (0.029)			
Bacteroidetes / Flavobacteriia	free-living	in situ	0.0903 (0.037)	0.246	0.861	-
		ambient	0.077 (0.017)			
		mid	0.076 (0.004)			
		high	0.08 (0.015)			
	attached	in situ	0.047 (0.009)	3.475	**0.079**	in situ/high: 0.081
		ambient	0.024 (0.018)			
		mid	0.022 (0.0005)			
		high	0.019 (0.006)			
Chloroflexi / SAR202	free-living	in situ	0.013 (0.002)	6.204	**0.017**	in situ/ambient: 0.021; ambient/mid: 0.027
		ambient	0.004 (0.002)			
		mid	0.013 (0.001)			
		high	0.01 (0.003)			
	attached	in situ	0.0004 (0.0001)	0.682	0.524	-
		ambient	0.0006 (0.0004)			
		mid	0.001 (0.0003)			
		high	0.0005 (0.0006)			
Crenarchaeota / Thaumarchaeota	free-living	in situ	0.058 (0.003)	3.017	**0.094**	-
		ambient	0.039 (0.014)			
		mid	0.075 (0.01)			
		high	0.073 (0.027)			
	attached	in situ	0.0007 (0.0002)	2.607	0.133	-
		ambient	0.002 (0.002)			
		mid	0.004 (0.0005)			
		high	0.001 (0.001)			

Euryarchaeota / Thermoplasmata	free-living	in situ	0.028 (0.001)	5.313	**0.026**	in situ/ambient: 0.023; ambient/mid: 0.074; ambient/high: 0.093
		ambient	0.008 (0.007)			
		mid	0.024 (0.002)			
		high	0.023 (0.01)			
	attached	in situ	0.0007 (0.0001)	0.817	0.524	-
		ambient	0.001 (0.0006)			
		mid	0.001 (1.37e-5)			
		high	0.001 (0.001)			
Planctomycetes / OM190	free-living	in situ	0.001 (0.0001)	5.461	**0.024**	in situ/ambient: 0.098; ambient/mid: 0.025; ambient/high: 0.049
		ambient	0.0006 (0.0003)			
		mid	0.001 (0.0001)			
		high	0.001 (0.0002)			
	attached	in situ	0.005 (0.002)	1.539	0.286	-
		ambient	0.007 (0.002)			
		mid	0.014 (0.004)			
		high	0.007 (0.008)			
Planctomycetes / Plamctomycetia	free-living	in situ	0.012 (0.002)	1.147	0.387	-
		ambient	0.014 (0.003)			
		mid	0.018 (0.004)			
		high	0.016 (0.004)			
	attached	in situ	0.004 (0.002)	1.909	0.216	-
		ambient	0.015 (0.006)			
		mid	0.01 (0.001)			
		high	0.009 (0.007)			
(Eukaryotes) Plastid	free-living	in situ	0.042 (0.009)	7.166	**0.011**	in situ/ambient: 0.0073
		ambient	0.007 (0.005)			
		mid	0.025 (0.007)			
		high	0.026 (0.012)			
	attached	in situ	0.173 (0.059)	1.576	0.278	-
		ambient	0.127 (0.065)			
		mid	0.307 (0.008)			
		high	0.152(0.154)			
Proteobacteria / Alphaproteobacteria	free-living	in situ	0.098 (0.023)	0.734	0.56	-
		ambient	0.117 (0.038)			
		mid	0.125 (0.026)			
		high	0.131 (0.022)			
	attached	in situ	0.012 (0.007)	2.882	0.112	in situ/high: 0.083
		ambient	0.0209 (0.006)			
		mid	0.019 (0.0002)			
		high	0.026 (0.004)			
Proteobacteria / Betaproteobacteria	free-living	in situ	0.024 (0.007)	0.256	0.854	-
		ambient	0.028 (0.008)			
		mid	0.027 (0.004)			
		high	0.029 (0.006)			
	attached	in situ	0.001 (0.0008)	1.091	0.413	-
		ambient	0.002 (0.001)			
		mid	0.004 (0.0003)			
		high	0.002 (0.002)			

Proteobacteria / Deltaproteobacteria	free-living	in situ	0.126 (0.026)	0.77	0.542	-
		ambient	0.109 (0.016)			
		mid	0.106 (0.014)			
		high	0.109 (0.007)			
	attached	in situ	0.021 (0.011)	1.07	0.42	-
		ambient	0.037 (0.023)			
		mid	0.053 (0.0009)			
		high	0.027 (0.027)			
Proteobacteria / Gammaproteobacteria	free-living	in situ	0.334 (0.065)	2.43	0.14	-
		ambient	0.437 (0.102)			
		mid	0.312(0.058)			
		high	0.306 (0.018)			
	attached	in situ	0.677 (0.103)	0.813	0.525	-
		ambient	0.623 (0.127)			
		mid	0.445 (0.005)			
		high	0.622 (0.267)			
Proteobacteria / Other	free-living	in situ	0.052 (0.011)	0.819	0.518	-
		ambient	0.049 (0.009)			
		mid	0.057 (0.005)			
		high	0.058 (0.004)			
	attached	in situ	0.01 (0.004)	0.207	0.888	-
		ambient	0.012 (0.004)			
		mid	0.011 (0.001)			
		high	0.011 (0.0008)			
SAR406 / AB16	free-living	in situ	0.034 (0.009)	5.686	**0.022**	in situ/ambient: 0.024; ambient/mid: 0.037
		ambient	0.012 (0.007)			
		mid	0.032 (0.002)			
		high	0.025 (0.007)			
	attached	in situ	0.0004 (0.0002)	0.346	0.793	-
		ambient	0.0007 (0.0006)			
		mid	0.001 (0.0002)			
		high	0.0009 (0.001)			
Verrucomicrobia / Verrucomicrobiae	free-living	in situ	0.003 (0.001)	4.141	**0.047**	in situ/ambient: 0.039
		ambient	0.014 (0.006)			
		mid	0.008 (0.001)			
		high	0.011 (0.003)			
	attached	in situ	0.002 (0.001)	1.866	0.223	-
		ambient	0.014 (0.011)			
		mid	0.007 (0.0005)			
		high	0.005 (0.004)			

Supplementary Table 3. Relative abundance summary and statistics of the main Gammaproteobacteria clades. Main Gammaproteobacteria clades were defined as those with mean relative abundances >0.5% in at least one sample.

Taxonomic group	Size fraction	Sample	Mean relative abundance (s.d.)	ANOVA F-value	ANOVA P-value	Adjusted P-value (Tukey's HSD test)
Alteromonadales	free-living	in situ	0.101 (0.043)	3.929	**0.054**	in situ/ambient: 0.079; ambient/mid: 0.091; ambient/high: 0.094
		ambient	0.292 (0.128)			
		mid	0.108 (0.079)			
		high	0.109 (0.04)			
	attached	in situ	0.433 (0.168)	0.633	0.616	-
		ambient	0.574 (0.144)			
		mid	0.391 (0.008)			
		high	0.581 (0.291)			
HTCC2188	free-living	in situ	0.0234 (0.006)	3.935	**0.053**	ambient/mid: 0.055; ambient/high: 0.099
		ambient	0.0131 (0.008)			
		mid	0.0268 (0.002)			
		high	0.025 (0.001)			
	attached	in situ	0.001 (0.001)	0.178	0.907	-
		ambient	0.002 (0.001)			
		mid	0.002 (0.0002)			
		high	0.001 (0.001)			
Oceanospirillales	free-living	in situ	0.107 (0.022)	1.45	0.298	-
		ambient	0.081 (0.025)			
		mid	0.117 (0.016)			
		high	0.108 (0.023)			
	attached	in situ	0.098 (0.033)	15.178	**0.001**	in situ/ambient: 0.004; in situ/mid: 0.007; in situ/high: 0.002
		ambient	0.014 (0.008)			
		mid	0.013 (0.002)			
		high	0.008 (0.007)			

Pseudomonadales	free-living	in situ	0.045 (0.073)	1.035	0.427	-
		ambient	0.0001 (0.0001)			
		mid	0.0007 (0.0007)			
		high	0.005 (0.008)			
	attached	in situ	0.113 (0.108)	2.58	0.136	-
		ambient	0.001 (0.001)			
		mid	0.005 (0.001)			
		high	0.006 (0.006)			
Xanthomonadales	free-living	in situ	0.002 (0.0006)	2.252	0.159	-
		ambient	0.003 (0.0008)			
		mid	0.003 (0.0006)			
		high	0.003 (0.0009)			
	attached	in situ	0.0009 (0.0002)	2.49	0.144	-
		ambient	0.004 (0.001)			
		mid	0.003 (0.0002)			
		high	0.002 (0.002)			
Other Gamma-proteobacteria	free-living	in situ	0.048 (0.006)	1.535	0.278	-
		ambient	0.041 (0.005)			
		mid	0.048 (0.003)			
		high	0.048 (0.004)			
	attached	in situ	0.027 (0.006)	0.888	0.492	-
		ambient	0.024 (0.006)			
		mid	0.024 (0.001)			
		high	0.019 (0.007)			

Supplementary Table 4. NRI (net-relatedness index) summary and statistics for the most abundant clades (defined as those with mean relative abundances >1% in at least one sample).

Taxonomic group	Size fraction	Sample	Mean NRI (s.d.)	ANOVA F-value	ANOVA P-value	Adjusted P-value (Tukey's HSD test)
Actinobacteria / Acidimicrobiia	free-living	in situ	-0.247 (0.157)	2.954	**0.097**	-
		ambient	1.241 (0.903)			
		mid	0.215 (0.454)			
		high	0.895 (0.878)			
	attached	in situ	1.497 (0.467)	10.238	**0.005**	ambient/high: 0.02; in situ/high: 0.091
		ambient	1.929 (0.625)			
		mid	-0.007 (0.089)			
		high	0.451 (0.309)			
Actinobacteria / Actinobacteria	free-living	in situ	3.763 (1.417)	0.23	0.872	-
		ambient	4.637 (1.481)			
		mid	4.325 (1.938)			
		high	3.702 (1.629)			
	attached	in situ	1.167 (1.881)	0.306	0.82	-
		ambient	1.138 (3.025)			
		mid	0.374 (1.887)			
		high	2.229 (1.563)			
Bacteroidetes / Flavobacteriia	free-living	in situ	2.774 (0.512)	1.142	0.388	-
		ambient	3.367 (0.346)			
		mid	2.921 (0.184)			
		high	2.979 (0.505)			
	attached	in situ	0.258 (0.896)	2.503	0.143	-
		ambient	-1.784 (0.539)			
		mid	-2.775 (0.589)			
		high	-1.691 (2.160)			
Chloroflexi / SAR202	free-living	in situ	1.412 (0.116)	0.218	0.88	-
		ambient	1.318 (0.497)			
		mid	1.521 (0.257)			
		high	1.438 (0.235)			
	attached	in situ	-0.047 (0.506)	4.764	**0.049**	in situ/mid: 0.037
		ambient	1.341 (0.883)			
		mid	2.432 (0.817)			
		high	1.046 (0.696)			

Crenarchaeota / Thaumarchaeota	free-living	in situ	1.778 (0.014)	8.238	**0.007**	in situ/high: 0.015; in situ/mid: 0.039; in situ/ambient: 0.009
		ambient	1.857 (0.017)			
		mid	1.837 (0.024)			
		high	1.849 (0.027)			
	attached	in situ	1.116 (1.201)	0.479	0.706	-
		ambient	1.719 (0.127)			
		mid	1.742 (0.005)			
		high	1.449 (0.513)			
Euryarchaeota / Thermoplasmata	free-living	in situ	0.080 (0.409)	0.536	0.669	-
		ambient	-0.007 (0.497)			
		mid	0.005 (0.241)			
		high	0.311 (0.125)			
	attached	in situ	1.812 (1.113)	1.233	0.376	-
		ambient	0.834 (0.528)			
		mid	0.638 (0.444)			
		high	1.087 (0.496)			
Planctomycetes / OM190	free-living	in situ	2.948 (0.647)	0.369	0.777	-
		ambient	2.401 (1.601)			
		mid	3.085 (0.101)			
		high	2.973 (0.238)			
	attached	in situ	0.417 (0.027)	0.669	0.597	-
		ambient	1.191 (1.600)			
		mid	1.639 (1.643)			
		high	1.439 (0.412)			
Planctomycetes / Plamctomycetia	free-living	in situ	3.167 (0.060)	0.0983	0.958	-
		ambient	3.149 (0.130)			
		mid	3.150 (0.118)			
		high	3.124 (0.055)			
	attached	in situ	1.174 (0.374)	2.308	0.163	-
		ambient	0.376 (2.451)			
		mid	0.302 (0.838)			
		high	-1.757 (0.761)			
(Eukaryotes) Plastid	free-living	in situ	4.077 (0.173)	0.989	0.445	-
		ambient	3.557 (1.298)			
		mid	4.431 (0.370)			
		high	4.361 (0.235)			
	attached	in situ	2.826 (0.921)	0.506	0.689	-
		ambient	2.221 (0.393)			
		mid	2.681 (0.688)			
		high	2.252 (0.772)			

Proteobacteria / Alphaproteobacteria	free-living	in situ	0.235 (0.812)	1.479	0.291	-
		ambient	2.139 (1.886)			
		mid	0.724 (0.546)			
		high	1.422 (1.039)			
	attached	in situ	2.523 (2.347)	0.716	0.572	-
		ambient	1.580 (1.215)			
		mid	-0.312 (0.973)			
		high	0.719 (3.212)			
Proteobacteria / Betaproteobacteria	free-living	in situ	2.148 (0.582)	2.448	0.138	-
		ambient	1.573 (0.225)			
		mid	1.401 (0.223)			
		high	1.477 (0.354)			
	attached	in situ	0.021 (1.285)	1.135	0.398	-
		ambient	0.903 (0.761)			
		mid	-0.198 (0.627)			
		high	0.898 (0.504)			
Proteobacteria / Deltaproteobacteria	free-living	in situ	4.462 (0.176)	4.066	**0.049**	ambient/mid: 0.089
		ambient	4.506 (0.080)			
		mid	3.941 (0.186)			
		high	4.037 (0.417)			
	attached	in situ	1.959 (0.952)	0.762	0.549	-
		ambient	2.364 (0.205)			
		mid	2.634 (0.124)			
		high	1.288 (1.791)			
Proteobacteria / Gamma-proteobacteria	free-living	in situ	5.374 (0.622)	1.157	0.383	-
		ambient	3.744 (0.831)			
		mid	4.847 (1.399)			
		high	4.485 (1.347)			
	attached	in situ	3.671 (0.126)	3.793	**0.066**	in situ/high: 0.095
		ambient	4.182 (0.719)			
		mid	5.049 (0.057)			
		high	4.936 (0.722)			
Proteobacteria / Other	free-living	in situ	1.682 (0.152)	11.679	**0.002**	ambient/high: 0.006; ambient/mid: 0.015; in situ/ambient: 0.003
		ambient	1.270 (0.103)			
		mid	1.583 (0.036)			
		high	1.638 (0.021)			
	attached	in situ	3.421 (0.278)	0.517	0.683	-
		ambient	1.338 (0.523)			
		mid	1.433 (0.569)			
		high	2.458 (4.205)			

SAR406 / AB16	free-living	in situ	1.404 (0.035)	0.683	0.586	-
		ambient	1.365 (0.220)			
		mid	1.390 (0.069)			
		high	1.261 (0.138)			
	attached	in situ	0.736 (1.820)	2.263	0.181	-
		ambient	-0.939 (0.049)			
		mid	1.725 (0.489)			
		high	0.417 (1.131)			
Verrucomicrobia / Verrucomicrobiae	free-living	in situ	1.991 (0.064)	10.882	**0.003**	in situ/high: 0.03; in situ/mid: 0.048; in situ/ambient: 0.002
		ambient	2.272 (0.0860)			
		mid	2.152 (0.052)			
		high	2.168 (0.022)			
	attached	in situ	1.072 (0.979)	0.549	0.664	-
		ambient	1.536 (0.1701)			
		mid	1.092(0.138)			
		high	1.462 (0.191)			

2. Supplementary Figures

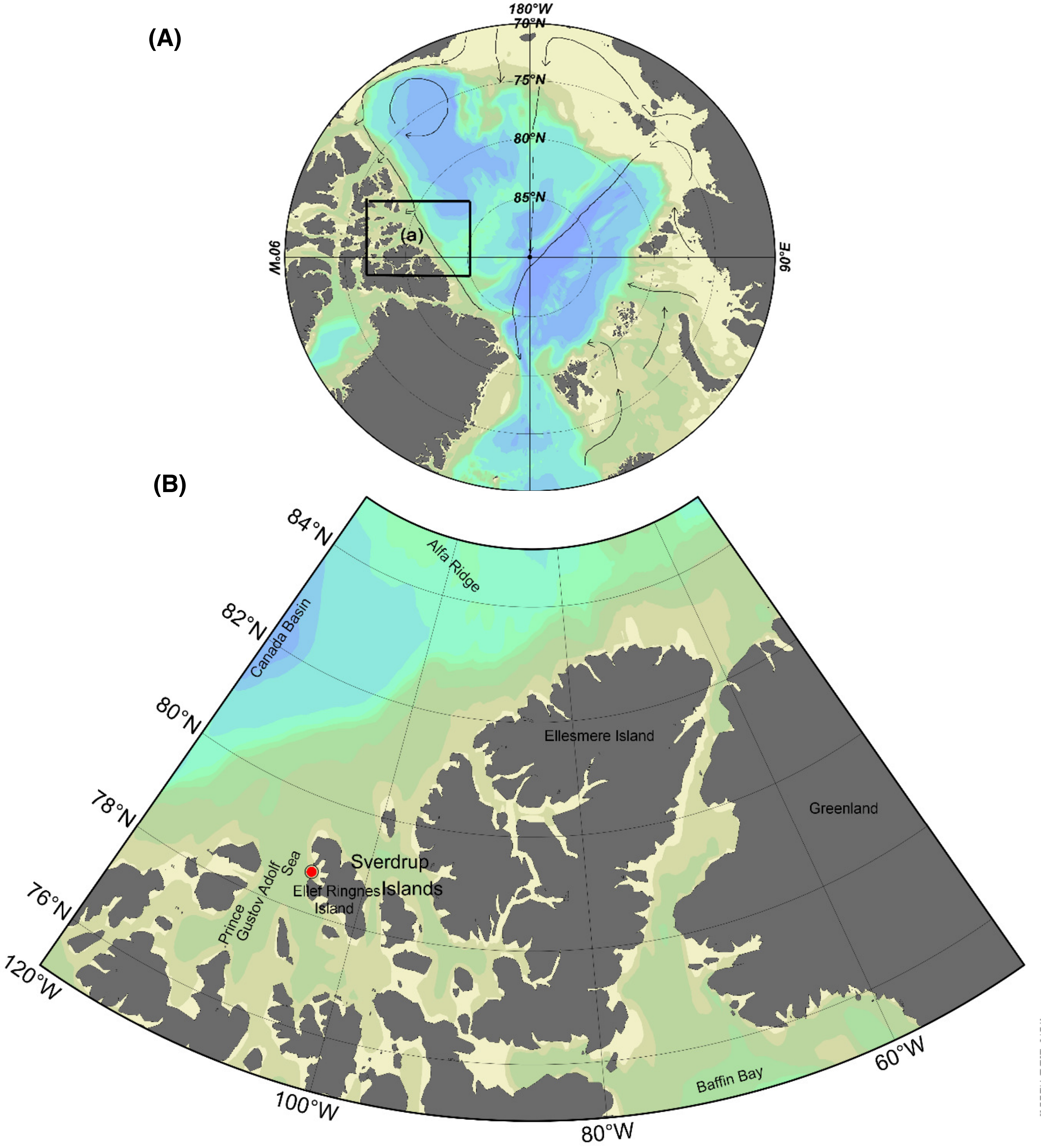

Supplementary Figure 1

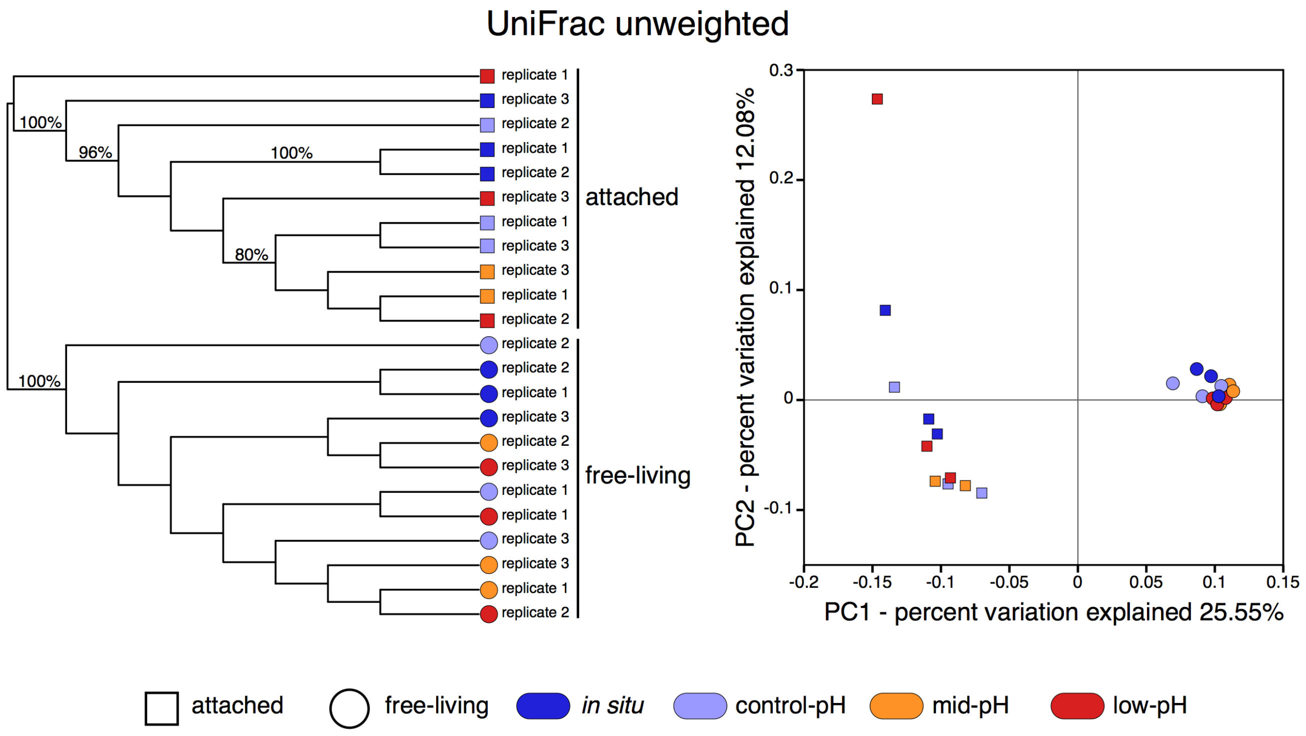

Supplementary Figure 2

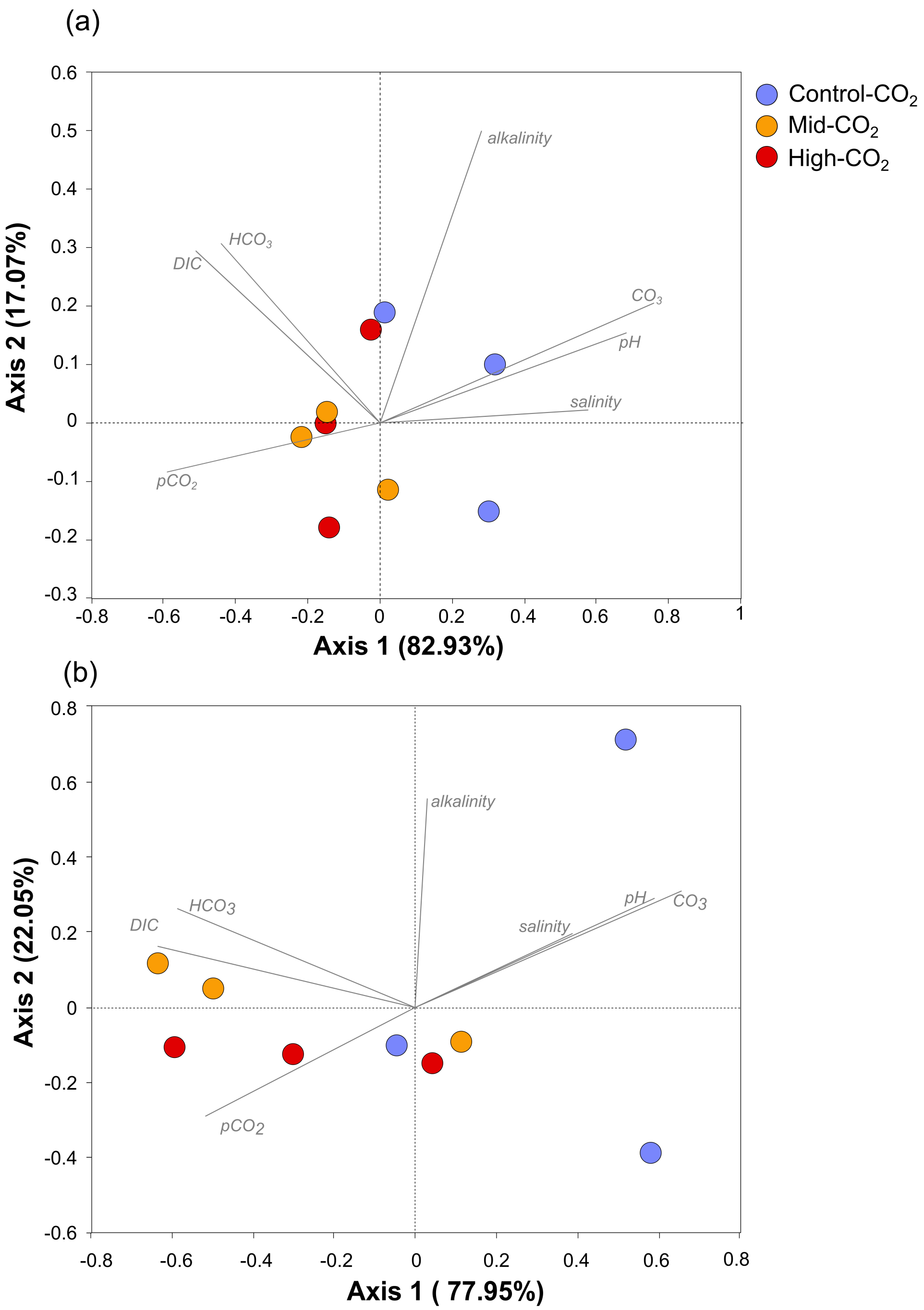

Supplementary Figure 3

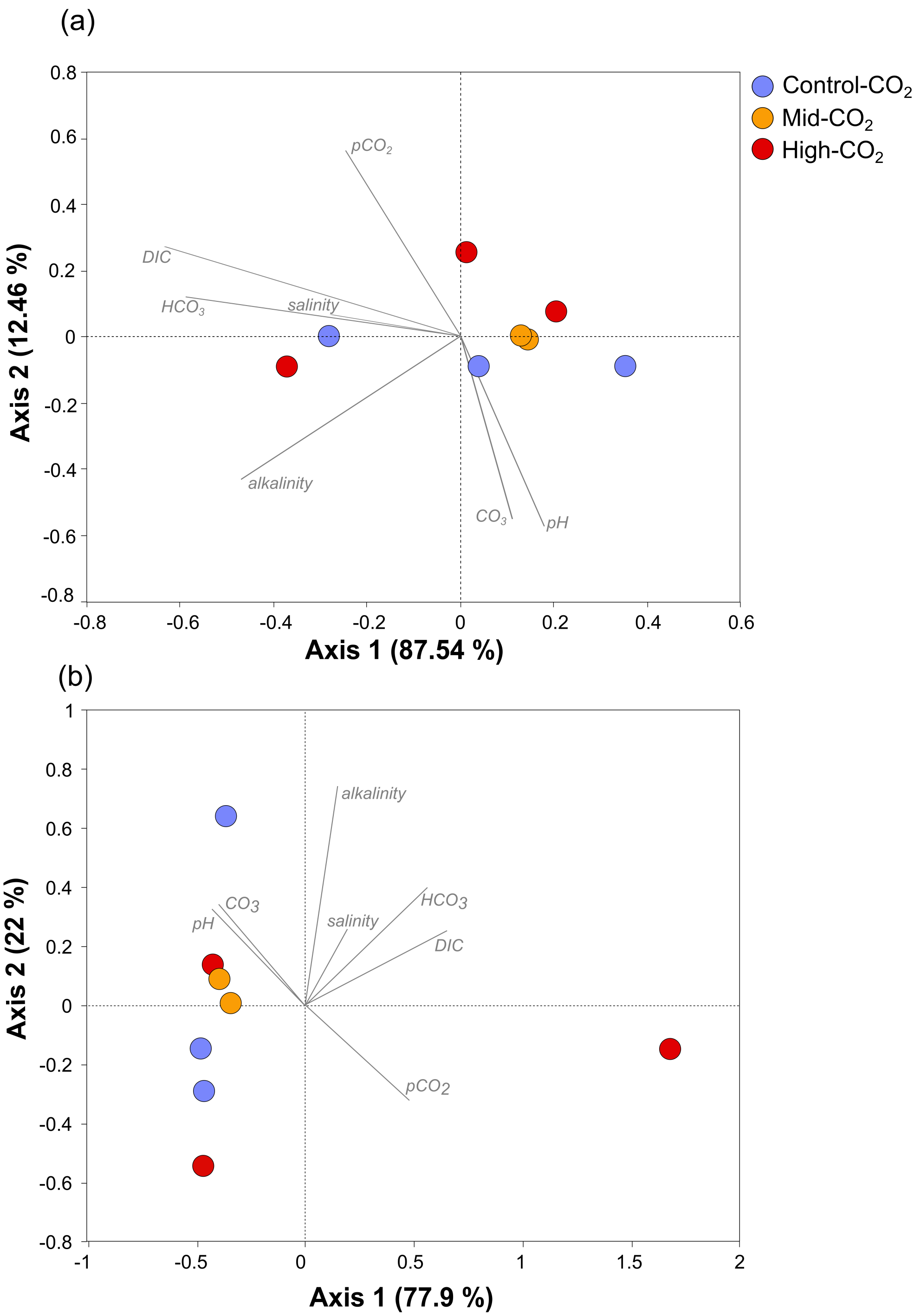

Supplementary Figure 4
